# Performance of Fetal Cardiac Volume Derived from VOCAL (Virtual Organ Computer-Aided AnaLysis) in Predicting Hemoglobin (Hb) Bart’s Disease

**DOI:** 10.3390/jcm10204651

**Published:** 2021-10-11

**Authors:** Keooudone Thammavong, Suchaya Luewan, Theera Tongsong

**Affiliations:** Department of Obstetrics and Gynecology, Faculty of Medicine, Chiang Mai University, Chiang Mai 50200, Thailand; keooudonet@gmail.com (K.T.); suchaya.l@cmu.ac.th (S.L.)

**Keywords:** cardiac volume, fetus, hemoglobin Bart’s disease, spatiotemporal image correlation (STIC), ultrasound, virtual organ computer-aided anaLysis (VOCAL)

## Abstract

Objective: To determine the performance of fetal cardiac volume (CV) in the detection of fetal Hb Bart’s disease among fetuses at risk at 18–22 weeks of gestation and to compare the performance with those of cardiothoracic diameter ratio (CTR) and middle cerebral artery peak systolic velocity (MCA-PSV). Methods: Fetuses at risk of Hb Bart’s disease between 18 and 22 weeks of gestation prospectively underwent echocardiography with acquisition of the volume datasets (VDS) of fetal heart, using 4D-cardiac STIC. Subsequently, off-line analysis was blindly performed to measure cardiac volume using the VOCAL technique. Results: A total of 502 fetuses at risk meeting the inclusion criteria were included in the analysis, consisting of 117 (23.3%) fetuses with Hb Bart’s disease and 385 (76.7%) unaffected fetuses. The mean (±SD) gestational age at the time of ultrasound examination was 19.70 ± 1.3 weeks. In predicting fetal Hb Bart’s disease, CV, using a cut-off Z-score of 1.7, had a sensitivity of 94.9% and specificity of 94.0%. The performance of CV was slightly better than that of CTR but very superior to that of MCA-PSV (areas under curve: 0.988, 0.974 and 0.862, respectively). Conclusions: Fetal CV has a very high performance in predicting fetal Hb Bart’s disease at mid-pregnancy, comparable with CTR and much better than MCA-PSV.

## 1. Introduction

Hb Bart’s disease is the most common cause of fetal anemia and hydrops fetalis in South-East Asia [1,2,3]. The affected fetuses are inevitably stillborn or die shortly after birth. If early termination is not performed, most affected pregnancies will develop severe preeclampsia, dystocia due to a large hydropic fetus and postpartum hemorrhage secondary to enlarged placenta. Accordingly, we have a national policy of routine thalassemia screening to identify the fetuses at risk for prenatal diagnosis [4], either DNA analysis via chorionic villi sampling and amniocentesis or fetal blood analysis via cordocentesis, leading to early detection and termination of pregnancy before development of hydrops fetalis and serious maternal complications. In our setting, all couples of affected fetuses had the pregnancies terminated. Bone marrow transplantation or gene therapy is only investigational and currently not available for this disease.

In the past three decades, ultrasound assessment of fetal anemia has been extensively studied, particularly anemia caused by isoimmunization in the Western world and hemoglobin (Hb) Bart’s disease in the East. The most commonly used ultrasound parameters are cardio-thoracic diameter ratio (CTR) and middle cerebral artery peak systolic velocity (MCA-PSV). In our practice, we have used both CTR and MCA-PSV measurements as screening tests for predicting Hb Bart’s disease among fetuses at risk [5,6,7]. Though CTR seems to be superior to MCA-PSV, the combination of both parameters can result in better performance.

Physiologically, in response to anemia, a fetus increases its cardiac size, represented by increased CTR, as well as develops hypervolemia to increase cardiac output for tissue oxygen perfusion; additionally, it increases blood flow velocity, represented by MCA-PSV, because of low viscosity. In clinical practice, both ultrasound parameters are very helpful in the assessment of fetal anemia.

Generally, fetal cardiac size is usually evaluated by cardiac diameter, especially CTR, cardiac circumference and cardiac area. CTR is the most commonly used ultrasound parameter for the evaluation of fetal cardiac size due to its high efficacy, ease of use and reproducibility [5,8,9]. However, cardiac volume (CV) can better represent the whole cardiac size than cardiac diameter, circumference and area, which include only one or two dimensions of the heart. Therefore, CV measurement is theoretically more accurate in measuring cardiac size and may lead to a more effective evaluation of fetal anemia caused by any disorders.

Nevertheless, until recently, estimation of cardiac volume was not practical in clinical use since it was technically difficult, labor intensive and time-consuming. However, with the current advancements in 4D-ultrasound technology of cardiac spatiotemporal image correlation (STIC), a volume dataset of a virtual heart can be simply manipulated in all axes to display the heart in the three orthogonal planes at the same time, making it easier to trace along the heart borders in all dimensions. Together with the function of the Virtual Organ Computer-aided AnaLysis (VOCAL) technique, the CV is readily calculated in a semi-automated function. Additionally, the volume datasets, with the virtual heart inside, can be acquired in a short time and digitally stored for subsequent off-line analysis, for the convenience of the sonographers. Additionally, the intraobserver and interobserver variations in CV measurement have been demonstrated to be in good agreement, with an ICC of 0.876 (95% CI 0.801–0.890, *p* < 0.001) and 0.767 (95% CI 0.638–0.854, *p* < 0.001), respectively [10]. This is also supported by the Bland–Altman plot [10]. Because there are no previous studies on the effectiveness of CV in the evaluation of fetal anemia, we conducted this study primarily to determine the performance of CV measurement in the detection of fetal anemia, using Hb Bart’s disease as a study model. The secondary objective is to compare the diagnostic indices of CV measurement with those of CTR and MCA-PSV measurements.

## 2. Materials and Methods

The study design is a diagnostic test study involving pregnant women at risk of fetal Hb Bart’s disease; it was conducted at Maharaj Nakorn Chiang Mai Hospital, a tertiary and medical teaching center. The study was ethically approved by the institutional review board (Faculty of Medicine, Chiang Mai University; Study Code: OBG-2563-07622/Research ID: 7622), and the participants provided written informed consent.

Under the project of prenatal control of severe thalassemia in our population [4], supported by the Thailand Research Fund, the prospective database of pregnant women at risk of having fetuses with Hb Bart’s disease was developed. The baseline characteristics of the women were obtained and recorded prospectively. All pregnancies underwent standard ultrasound examination and fetal echocardiography, using Cardio-STIC to acquire the volume datasets of the fetal heart for subsequent analysis. All of the volume datasets were acquired by the MFM (maternal-fetal medicine) fellows or MFM staff members, using Voluson E8 and Voluson E10 machine (GE Healthcare Ultrasound, Milwaukee, WI, USA), equipped with transabdominal 2-to 4-MHz curvilinear transducers. The protocol used for the acquisition of cardiac volume was as follows: The acquisition of STIC volumes was performed after real-time echocardiography. The standardized acquisition plane was the four-chamber view, preferably by an apical approach. All volumes were acquired using Speckle Reduction Imaging (by default set to SRI-II 4) and using CrossXBeam (CompoundResolution Imaging: CRI). The acquisition angle was 25° (usually set approximately 5° greater than the gestational week), but the angle can always be adjusted, depending on fetal size. Typically this is the minimum acquisition angle that will include only the region of interest. The acquisition time was set for 10–12.5 s. The participants were asked to hold their breath during volume acquisition (lasting 10–12.5 s) which was performed in the absence of fetal movement.

Additionally, CTR and MCA-PSV were also measured during ultrasound examination, using real-time 2D-ultrasound. The CTR and MCA-PSV measurements followed the well-established techniques published elsewhere [11,12]. All ultrasound scans were performed prior to the invasive diagnosis (cord blood sampling).

The database was accessed to retrieve all consecutive data records of the pregnant women at risk of having affected fetuses who met the inclusion criteria, enrolled between January 2012 and December 2020. The inclusion criteria are as follows: (1) singleton pregnancy; (2) gestational age between 18 and 22 weeks of pregnancy, based on sonographic dating in the first half of pregnancy; (3) at risk of having fetuses with Hb Bart’s disease (the couple, i.e., both parents, were a carrier of alpha-thalassemia-1 gene: αα/--SEA), recruited from our thalassemia screening program [4]; (4) known definite diagnosis of fetal Hb Bart’s disease or unaffected fetuses, based on fetal blood sampling (cordocentesis), using HPLC (high performance liquid chromatography) for fetal hemoglobin typing as a gold standard in diagnosis of Hb Bart’s disease. The exclusion criteria consist of the following: (1) unknown final diagnosis or loss-to-follow up; (2) poor quality of the volume datasets (validated by one of the authors: TT); (3) fetal anomalies or chromosomal abnormalities; (4) fetal anemia associated with other causes; for examples: TORCH infection, Rh alloimmunization, etc.

The details of ultrasound scans, including still images, 2D biometric measurement values, and cine video loops, and identification data were recorded automatically as DICOM files in the ultrasound hard disk at the time of scans. All volume datasets were subsequently assigned to one (KT) of the authors for cardiac volume (CV) measurements. The author (KT) who performed CV measurements was blinded to the final diagnosis of the fetuses.

### 2.1. Cardiac Volume Measurements

**Figure 1:**
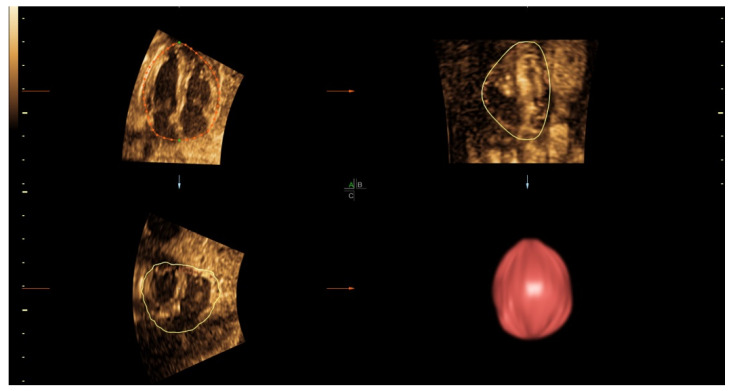
An example of cardiac volume measurement at 20 weeks of gestation, using VOCAL techniques, with rendering to show the volume of 10.52 mL (A/B/C is the name of each panel in multiplanar view).

The volume datasets were analyzed using 4D-View software version 14 (GE Medical Systems, Zipf, Austria). Each dataset, which contained the virtual heart pulsating along one cardiac cycle from start-systole to end-diastole, was firstly loaded on the multiplanar view and then maneuvered to display the best apical four-chamber view. On off-line analysis, the volume datasets underwent image processing such as speed or brightness adjustment to optimize tissue contrast resolution as appropriate for analysis. By default, the gray chroma map was set to sepia and SRI-II 4 was initially set for both 2D and 3D images. The measurement of CVs was performed on the end-diastolic frame, using the Virtual Organ Computer-aided AnaLysis (VOCAL) function of 4D-View (Figure 1). The Trace option on the VOCAL II-Define Contour panel was then activated, and the rotation step of 6° was chosen. The selected image was enlarged to occupy about three-fourths of the monitor. The image was adjusted for color, contrast and SRI to optimize the cardiac border. On measurement, the reference dots were repositioned and traced along the cardiac outline. The rotation axis was defined by setting a vertical line from the outer border of the cardiac base to the outer border of the apex. The drawn dot line was finely adjusted manually to fit the contour of heart border. The first plane of rotation was the best four-chamber view at the end-diastole. A total of 30 sliced images were rotated in sequence around the fixed vertical axis, with a step of 6° rotation from the previous slice. After the 30 contours of rotational images were successfully measured, the built-in computing function was activated to automatically compute the cardiac volume. All of the CV measurements were performed by the same author (KT).

### 2.2. Statistical Analysis

The primary measure is the performance of CV in predicting fetal Hb Bart’s disease. The secondary measures are the comparisons of the performance of CV with those of CTR and MCA-PSV measurements. To compare the diagnostic performances of the three sonographic parameters, the statistical procedures were as follows: the Z-scores of each actual CV and CTR measurement value were calculated, based on the reference models, previously reported [10,12]; Z-score = (Actual measured value-Predicted value from the model)/Predicted standard deviation. The receiver operating characteristic (ROC) curve of CV Z-scores, CTR Z-scores and MCA-PSV MoMs (multiples of median) in predicting the affected fetuses were constructed. The areas under curve (AUC) of the three sonographic parameters were compared, using Z-test. According to the best cut-off values of the three parameters derived from the ROC curves, the diagnostic indices (sensitivity, specificity, positive predictive value and negative predictive value) with 95% CI were determined. A *p*-value of less than 0.05 was considered as statistical significance. All analyses were performed using the statistical package for the social sciences (SPSS) software version 26.0 (IBM Corp. Released 2019. IBM SPSS Statistics for Windows, Version 26.0, IBM Corp, Armonk, NY, USA).

### 2.3. Sample Size Estimation

As a diagnostic test study, given a sensitivity of approximately 95% according to our previous study on CTR [7], which was relatively equivalent to this study, the study needed a sample size of at least 73 pregnancies with fetal Hb Bart’s disease or 292 pregnancies at risk (25% chance of affected), at a 95% confident interval and allowable error of 0.05.

## 3. Results

**Figure 2:**
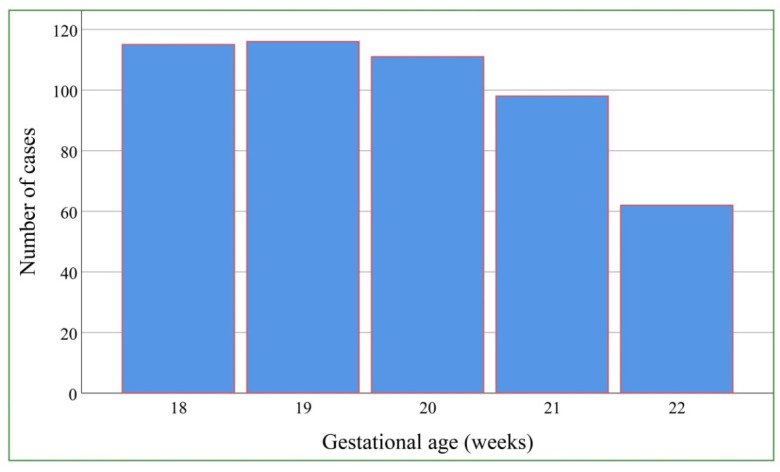
Distribution of gestational age at the time of ultrasound diagnosis.

During the period of recruitment (2012–2020), a total of 554 pregnancies at risk of fetal Hb Bart’s disease underwent fetal echocardiography with successful volume dataset acquisition. Of them, 32 were excluded because of poor quality volume datasets, 20 cases were excluded because the volume datasets did not include the whole heart, and the remaining 502 sets meeting the inclusion criteria were available for analysis, consisting of 117 (23.3%) affected fetuses (Hb Bart’s disease) and 385 (76.7%) unaffected fetuses (non-Hb Bart’s disease). All pregnant women with an affected fetus decided to terminate the pregnancy. Note that cordocentesis was performed in all cases, although the possibility of an earlier chorionic biopsy is a clinically available option and that there are preliminary data on non-invasive DNA, potentially to be implemented in the future. The demographic data of the pregnant women are presented in Table 1. Nearly all of the women (97.6%) were of Thai nationality. The mean (±SD) maternal age was 28.7 ± 6.1 years. The mean (±SD) gestational age at the time of ultrasound examination was 19.7 ± 1.3 weeks. The distribution of gestational age at the time of ultrasound diagnosis is presented in Figure 2.

**Figure 3:**
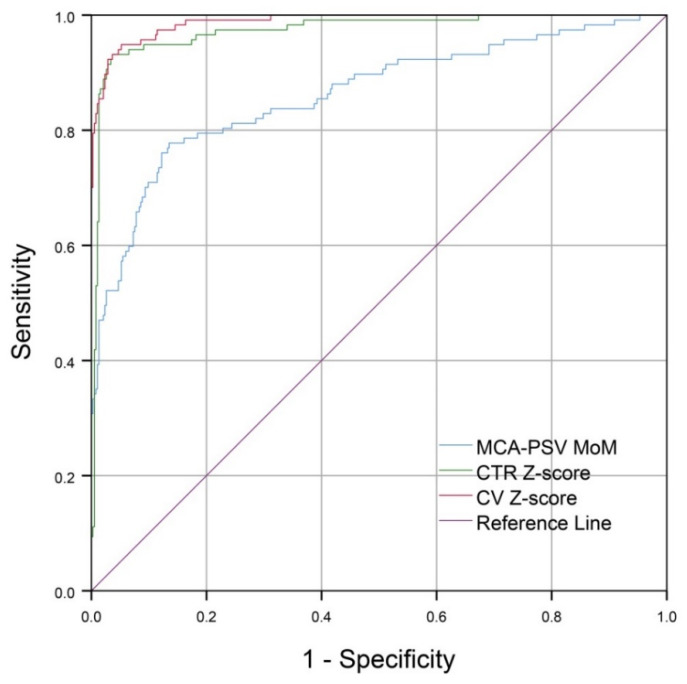
The ROC curves represent performances of CV Z-scores, CTR Z-scores and MCA-PSV MoMs in predicting the fetuses with Hb Bart’s disease.

The performance of CV Z-scores is slightly better than that of CTR Z-scores, but very superior to that of MCA-PSV MoM values; the areas under curve (AUC) are 0.988, 0.974, and 0.862, respectively. The mean ± SD Z-score of CV in the affected group was 3.08 ± 0.9 whereas that of the unaffected group was 0.01 ± 0.8 (*p*-value < 0.001). Z-test shows that the AUC of CV Z-scores is comparable to that of CTR Z-scores (*p*-value: 0.120; AUC difference: 0.015 (95% CI: −0.004 to 0.033)) but significantly different from that of MCA-PSV (*p*-value < 0.001; AUC difference: 0.126 (95% CI: −0.082 to 0.170)), as presented in Figure 3 and Table 2.

The diagnostic indices of CV, CTR and MCA-PSV are presented in Table 3. Based on the best cut-off values derived from the ROC Curves, CV Z-scores, using cut-off > 1.7, gave a sensitivity of 94.9% and specificity of 94.0%, slightly higher than CTR Z-scores, using cut-off > 1.3, which gave a sensitivity of 92.3% and a specificity of 96.6%. The best cut-off of MCA-PSV was 1.3 MoM, giving a sensitivity of 77.8% and specificity of 86.2%.

## 4. Discussion

In adaptation to anemic hypoxia, fetuses develop hypervolemia and increased cardiac size to increase cardiac output to maintain tissue oxygen perfusion. The earliest sonographic sign of fetuses affected with Hb Bart’s disease is increased cardiac size. Regarding fetuses with Hb Bart’s disease, usually associated with low Hb levels (3–8 g/dL) [5,7], CTR is the most commonly used sonographic marker [6,13] because of its high effectiveness and simplicity. Nevertheless, as mentioned earlier, CV might be superior to CTR because it includes all dimensions of the heart and theoretically better represents cardiac size than CTR does. Therefore, CV should hypothetically have greater performance than CTR in the prediction of the affected fetuses. However, our result suggests that the performance is comparable, though CV may be slightly better, as shown in the ROC curve. Accordingly, this study does not support the use of CV in daily practice, in spite of its high effectiveness, since it has more disadvantages than CTR in terms of simplicity, availability and time consumption. Nevertheless, this study provides evidence that CV is a new and highly effective sonographic marker in predicting fetal Hb Bart’s disease. Though it should not replace CTR, it may be useful as an adjunct, especially in cases of abnormal cardiac shape or contour or abnormal four-chamber view. We believe that CV can also be used in evaluation of fetuses with anemia due to other causes. Though CV is theoretically expected to have a higher performance, our evidence does not strongly support such a concept. It is possible that CV measurement is less accurate compared to CTR measurement. This is due to the fact that in the measurement of CTR, only one diameter is used and the landmark for cursor placement is usually clear and easier to identify, while in CV measurement, the cardiac contour for cursor tracing along the cardiac outline might not be very clear and is subject to more variations, leading to less accuracy in measurement. Since the advancement in 4D ultrasound technology is ongoing, we hope that, with future high resolution, differentiation between the cardiac borders and surrounding tissue will be improved, resulting in high accuracy in CV measurement. Importantly, volume dataset acquisition is simple, needs no expertise and is less-time consuming. Thus, cardiac volume datasets can be acquired anywhere and digitally sent instantaneously to MFM centers for CV calculation and interpretation by experts.

According to the ROC curves of the three parameters, CV has the best diagnostic performance, though comparable with/slightly better than CTR and much better than MCA-PSV, indicating that cardiac size is more sensitive than blood flow velocity in the detection of Hb Bart’s disease, which is usually associated with severe anemia. The main new insight provided by this study is that CV is a highly effective ultrasound parameter in the diagnosis of fetal anemia, using Hb Bart’s disease as a study model, and it might be useful in the detection of fetal anemia secondary to other causes, though this needs to be elucidated by future studies. Since CV is gestational age-dependent, the cut-off point using a date-independent Z-score rather than the absolute value of CV is more appropriate to define a normal or abnormal CV. Based on the ROC curve, this study shows that the cut-off Z-score of 1.7 gave the best diagnostic indices.

Another important insight gained from this study is that cardiac size, either CV or CTR, is much more sensitive than MCA-PSV in predicting fetal Hb Bart’s disease. Such a finding raises a question on why cardiac size has rarely been used for the evaluation of fetal anemia due to any causes other than Hb Bart’s disease. Moreover, a study comparing the effectiveness of the two markers in identifying fetal anemia due to other causes has never been published. In practice, most authors recommend MCA-PSV in predicting anemia among fetuses at risk of Rh isoimmunization, parvovirus B19, etc., and also monitor the progression of its severity or improvement after fetal treatment [11,14]. Although our finding showed the superiority of cardiac size to MCA-PSV, such a good performance may not be reproduced in the case of anemia caused by other disorders. Probably, hypoxia, either due to anemia or abnormal hemoglobin, rather than anemia itself, is a primary cause of an increase in cardiac size in order to increase cardiac output to preserve tissue oxygen perfusion. Our hypothesis is that many fetuses with Hb Bart’s disease have severe hypoxia due to low levels of hemoglobin, in spite of only mild anemia. In such cases, cardiac size first increases, while an increase in blood flow velocity is only subtle. However, this hypothesis needs to be confirmed by further studies with hemoglobin measurement together with cardiac size and MCA-PSV.

The limitations of this study include the following: (1) since fetal anemia in this study was uniformly caused by Hb Bart’s disease, the diagnostic performance of the three ultrasound parameters in the detection of fetal anemia might not be reproducible when the test involves cases of anemia secondary to other causes. (2) The severity of anemia was not routinely evaluated at the time of cordocentesis. Therefore, we could not correlate it with the degree of cardiomegaly and other cardiac function parameters. (3) All volume datasets were acquired by MFM specialists, and CV measurements were performed by the authors. Accordingly, the high effectiveness of the three parameters may not be reproducible in daily practice by general practitioners. (4) Cardio-STIC with VOCAL function is currently not available in most prenatal clinics. Additionally, CV measurements need more expertise and are time-consuming, though relatively simple and convenient for examiners to do off-line analysis. (5) The effect of experience levels of the examiners on diagnostic performance was not assessed. (6) The exact number of cases with feasibility of volume acquisition in actual practice could not be provided. Additionally, some cases were excluded because of poor image quality and incomplete acquisition. Most were associated with unfavorable fetal exposure, fetal active movement and thickened maternal abdominal wall. This may be partly improved by taking more time of examination or rescanning later and strictly adhering to the standard protocol of acquisition.

The strengths of this study consist of the following: (1) on VOCAL analysis, we used the greatest number of slices (images) per heart by rotating the volume dataset with the least angle (6°) at a time. Thus, the rendered cardiac volume is expected to most closely represent the real heart volume. (2) Since the author who measured CVs was blinded to the fetal diagnosis, bias in analysis of the volume datasets was probably low. (3) The comparisons of the predictive performance among the three parameters are expected to be highly reliable since the three sonographic markers were tested on the same fetuses, representing perfectly matched sets. (4) The large sample size or high power of test makes the conclusion more reliable.

While MCA-PSV is commonly used in the Western world for evaluation of fetuses at risk of anemia secondary to any causes, this study demonstrates that CV has a significantly higher performance than that of MCA-PSV in predicting fetal Hb Bart’s disease. Accordingly, we suggest that future studies should be performed to compare the effectiveness in predicting fetal anemia from other causes between MCA-PSV and cardiac size.

## 5. Conclusions

CV measurement with the VOCAL technique has high diagnostic indices in the detection of fetal Hb Bart’s disease. Its performance is comparable to CTR but obviously superior to MCA-PSV. At Z-score cut-off > 1.7, CV gives a sensitivity of 94.9% and a specificity of 94.0% in the detection of fetal Hb Bart’s disease. Finally, CV measurements may be superior to those of CTR in cases of anatomical variations of the cardiac structures, resulting in less reliability of CTR measurements.

## Figures and Tables

**Table 1:** Demographic data of the pregnant women.

Characteristics	N (%) or Means ± SD
Maternal age (mean ± SD)	28.7 ± 6.1
Gestational age (week) at diagnosis (mean ± SD)	19.7 ± 1.3
Parity	
Nulliparous	284 (56.6%)
Parous	218 (43.4%)
Age group	
Early adolescent (less than 16 years)	7 (1.4%)
Late adolescent (16–19 years)	24 (4.8%)
Reproductive age (20–34 years)	382 (76.1%)
Elderly (35 years or greater)	89 (17.7%)
Residency	
Chiang Mai	336 (66.9%)
Others	166 (33.1%)

**Table 2:** Comparisons of the areas under ROC curve of CV Z-scores, CTR-Z-scores and MCA-PSV MoMs in predicting fetal Hb Bart’s disease.

Ultrasound Parameters	AUC (95% CI)	*p*-Value
1. Cardiac volume (Z-scores)	0.988 (0.981–0.996)	<0.001
2. Cardiac-thoracic diameter ratio (Z-scores)	0.974 (0.957–0.990)	
3. MCA-PSV (MoMs)	0.862 (0.819–0.906)	

Z-test 1 vs. 2; AUC difference: 0.015 (95% CI: −0.04–0.033), *p*-value: =0.120. Z-test 1 vs. 3; AUC difference: 0.126 (95% CI: 0.082–0.170), *p*-value: <0.001.

**Table 3:** Diagnostic indices of cardiac volume (CV), cardiac-thoracic diameter ratio (CTR) and MCA-PSV in predicting fetal Hb Bart’s disease.

Ultrasound Parameters	Cut-Off Value	Sensitivity (95%CI)(*n*/N)	Specificity (95%CI)(*n*/N)	Positive Predictive Value (95%CI) (*n*/N)	Positive Predictive Value (95%CI) (*n*/N)
Cardiac volume (Z-scores)	>1.7	94.9% (90.9–98.9) (111/117)	94.0% (91.7–96.4) (362/385)	82.8% (76.5–89.2) (111/134)	98.4% (96.2–100.0) (362/368)
Cardiac-thoracic diameter ratio (Z-scores)	>1.3 *	92.3% (87.5–97.1) (108/117)	96.6% (94.8–98.4) (372/385)	89.3% (83.7–94.8) (108/121)	97.6% (94.9–100.0) (372/381)
MCA-PSV (MoMs)	>1.3	77.8% (70.2–85.3) (91/117)	86.2% (82.8–89.7) (332/385)	63.2% (55.3–71.1) (91/144)	92.7% (88.5–97.0) (332/358)

* Z-score 1.3 equivalent to CTR > 0.50 (18–20 weeks) and > 0.51 (21–22 weeks).

## Data Availability

The datasets analyzed during the current study are available from the corresponding author upon reasonable request.
